# Progress in Cancer

**Published:** 1903-12-26

**Authors:** 


### PROGRESS IN CANCER.

(*Concluded from page* 207.)

Attention is drawn by Johnson[5] to Otto Schmidt's specific treatment of cancer by means of a fluid containing the killed parasites, or a serum derived from horses or sheep which have been injected with such fluid. The parasites obtained by him showed various appearances; Schiller's, Russell's, and Plimmer's bodies being varieties of this one parasite. Injections are made beginning with $\frac{1}{100}$ mgr. in ·5 per cent. Ac. carbol. solution, gradually increased to a maximum of 1 centgrm. The injections are not made immediately into the growth but into any other part of the body subcutaneously. There is no reaction in non-cancerous, but always one in cancerous tumours, with the exception of cachectic persons whose blood may be supposed to be charged with anti-bodies. The reactions diminish as immunity is approached. In open growths secretion dries up and purulent discharge ceases; a crust forms and is cast off and with it superficial layers of the growth. This is repeated till the whole is removed. In the deeper tumours the growths become gangrenous and are cast off. Sufficient has now been done with this serum to show great promise.

That the normal epithelium of the trachea of an ox contains certain enzymes has been demonstrated by Harden and MacFadyen.[6] These are an invertase, an amylase, a maltase, and a proteolytic enzyme. Also they show that human cancerous tissue contain the following enzymes, viz., an invertase, a maltase, an amylase, proteolytic enzymes acting in acid and alkaline solutions respectively, a catalase and an oxidase together with traces of a lipase and possibly of a peroxidase.

Sir Felix Semon[7] in speaking of operations for malignant disease of the larynx records from his own experience that unless recurrence occurs within a year of the operation the word "cure" can safely be employed. If anything could be calculated, he says, to rouse sceptics from their present pessimistic attitude with regard to the advisability of operating upon cancers it ought to be the brilliant results obtained in cancer of the larynx, when the diagnosis has been made early, a suitable operation been selected, and no precious time been lost.

Professor Gluck[8] speaking on the same subject reports that he has had a series of 27 cases with only one death of complete laryngectomies for cancer of the larynx.

Dr. Moure[9] considers that tumours of the cords are much less malignant than those growing from other parts of the mucous membrane, and admit of better prognosis.

Sändberg[10] has studied 2,153 cases of cancer. Of these less than one-third were in male subjects, and one-half of these were located in the stomach (315 out of 623). About 1·71 per cent. of all male patients were affected by cancer, and 4·32 per cent. of women, out of a total number of patients of 71,839. As results after operation he gives that cancer of the stomach developed without metastases in 52·5 per cent. of the 229 cases in women; in 54·7 per cent. in the 315 male cases. Metastases were most common among cases of cancer in the liver and were found in 101 out of 483 cases of cancer of the uterus, and in 131 out of 288 cases of cancer of the mamma.

Having met with 12 cases of diabetes out of 366 cases of intestinal carcinoma, Boas[11] discusses the coincidence of these diseases. In these cases diabetes had existed from two months to seven years to the patients' knowledge: the amount of sugar varying from 1·2 per cent. to 5·3 per cent. In three cases operated upon one, being free from sugar at the time, recovered. A second died in coma, and the third stood the operation well, but died nine months later.

Carcinoma is affected by diabetes in the active stages: when the latter is latent the carcinoma appears to be rendered less rapid and malignant than usual. There seem to be two classes of cases as far as diabetes is concerned: (1) Those in which the glycosuria diminishes as soon as the carcinoma sets in; and (2) those in whom the diabetes is unaffected by the carcinoma. He recommends that in the treatment too rigid an animal diet should not be enforced, and that carbohydrates should be permitted in moderate amount.

As an aid in the diagnosis of carcinoma of the stomach, Salomon[12] suggests that the organ should be thoroughly washed out with normal salt solution again and again—no albuminous food having been taken for the previous 24 hours—and the washings tested for nitrogen. In the case of carcinoma, albumin will be found, whereas in other diseased conditions it will not be found to anything like the same extent. He measures the nitrogen by Kjehldahl's process and albumin by Esbach's test. When[13] the fluid shows a flaky turbidness or the nitrogen is over 20 mgrm. in 100 cc., then cancer may be suspected. Félix[14] considers that the important causes of cancer are traumatism, inflammation, and irritation. He denies the existence of the cancerous diathesis, except in so far as that in gouty subjects the depositions of urate crystals may determine cancer growth. He advocates as treatment the use of a caustic paste containing carbolic acid, zinc chlorate, iodol, etc., in preference to the knife. Regimen and diet are important in the prophylaxis of cancer.

According to Dr. Bashford,[15] as far as malignant growths are concerned, other than rodent ulcers, the results so far brought to his notice of such treatments as the Finsen light, high-frequency currents, and $x$-rays, do not establish the efficiency of any of these measures as curative agents in sarcoma and carcinoma. The whole question must be considered sub judice. Loeb[16] has frequently discovered cancers in the eyes of animals. He states that it is not uncommonly found in the Chicago stockyards, whereas only two external carcinomata have been noticed elsewhere. Carcinomata seem to be endemic in a certain farm in Wyoming, but no cases occur in the neighbourhood.

Jensen[17] has succeeded in transmitting through 22 generations of white mice by transplantation a tumour originally derived from a melano-sarcoma of a horse. There was pronounced carcinomatous structure, but no metastases. The tumours grew till the animals died of cachexia or ulceration of the testicles. The transplantations were successful in 40 to 50 per cent. of all attempts on white mice; but, with the exception of a few ordinary mice, they were unsuccessful in other animals. There was no evidence of parasitic action.

Feinberg[18] differentiates between the histological characters of the nuclear arrangement of the protozoa, the yeasts, and the "cancer bodies." In the protozoa he says there is a compact chromatin body with a surrounding halo of nuclear sap. In metazoa there is no nuclear network, nucleolus or nucleolar substance. In the yeasts there is no nuclear sap, but the distinction from protozoa has a weak link in the fact that trypanosomata have none either. He considers that in the epithelial cells of carcinomata there are bodies which must be considered to be sporozoa.

In a paper on the Endotheliomata Stuart McDonald[19] writes that it is now generally conceded that these tumours belong to the sarcomata rather than the true cancers. The question, he says, turns upon the difference between endothelium and epithelium. The flattened endothelial cell lining the wall of a blood-vessel is very different from that which has proliferated from it in tumour formation. This may be spindle-shaped, cuboidal, cylindrical, etc. They form distinct intercellular substance, and distinct connective tissue at times. In distinction from true cancers which they nearly resemble, it will be seen that at the periphery the epithelioid cells pass into the flat endothelial cells of the lymph vessel or spaces (Borst). The tendency is for the cells to occur in connective tissue spaces which they fill in every direction; so that the stroma forming the spaces and containing the vessels is surrounded in every direction by cells (Ribbert). There is more intimate connection between the fibrous stroma outside, and the cells in the space than in carcinoma, the fine fibrillæ actually pass in from the edge of the space between the endothelial cells (Borst). Blood-vessels may be seen next to and amongst the cells without intervening stroma, which is not the case in carcinomata (Waldeyer). Degenerative changes are extremely prone to occur, especially the hyaline, mucoid, and myxomatous, the hyaline degeneration affecting the walls of the blood-vessels and the myxomatous the stroma. Probably the great majority, he says, of these tumours arise from the endothelial lining of the lymphatic spaces, though at times growths are found arranged concentrically round a blood-vessel, strongly suggestive of their origin from the vascular endothelium, but it may be they arise from the perivascular lymphatics. There is a growing belief that many of the diffuse scirrhus growths of the stomach and intestines are really endothelial in origin. Many primary endotheliomata of the pleura have recently been described. In endotheliomata of the parotid and submaxillary glands there is often a stroma of extremely complex structure. Hyaline, fatty, myxomatous, sarcomatous and cartilaginous tissue may all be present, and the so called "mixed" tumours of the parotid and testicles may be of this nature. It would seem that these tumours are much more common than generally supposed.

[5] Brit. Med. Jour., Nov. 14. [6] Lancet, July 25. [7] Brit. Med. Jour., Oct. 31. [8] Ibid. [9] Ibid. [10] Jour. A.M.A., Aug. 22. [11] Brit. Med. Jour., Nov. 7. [12] Münchener Med. Wochensch, Aug. 11, 1903. [13] Jour. A.M.A., Sept. 12, 1903. [14] Brit. Med. Jour., Nov. 7. [15] Ibid., Aug. 15. [16] Jour. A.M.A., Oct. 3, 1903 [17] Ibid., Aug. 29. [18] Brit. Med. Jour., Nov. 7. [19] Scot. Jour. Med. Sci., July.

